# A numerical solution of a singular boundary value problem arising in boundary layer theory

**DOI:** 10.1186/s40064-016-1835-z

**Published:** 2016-02-27

**Authors:** Jiancheng Hu

**Affiliations:** College of Applied Mathematics, Chengdu University of Information Technology, Chengdu, 610225 China

**Keywords:** Falkner–Skan equation, Nonlinear boundary value problems, Newton’s method, Finite difference method

## Abstract

In this paper, a second-order nonlinear singular boundary value problem is presented, which is equivalent to the well-known Falkner–Skan equation. And the one-dimensional third-order boundary value problem on interval $[0,\infty )$ is equivalently transformed into a second-order boundary value problem on finite interval $[\beta , 1]$. The finite difference method is utilized to solve the singular boundary value problem, in which the amount of computational effort is significantly less than the other numerical methods. The numerical solutions obtained by the finite difference method are in agreement with those obtained by previous authors.

## Background

The well-known nonlinear third-order Falkner–Skan equation is one of the nonlinear two-point boundary value problem (BVP) on infinite intervals. This problem arises in the study of laminar boundary layers exhibiting similarity in fluid mechanics. The solutions of the one-dimensional third-order boundary-value problem described by the Falkner–Skan equation are the similarity solutions of the two-dimensional incompressible laminar boundary layer equations (Cheng 1977; Merkin 1980; Salama 2004; Postelnicu and Pop 2011; Mosayebidorcheh 2013).

Considering the following differential equation (Aly et al. 2003):

1 $$\begin{aligned} f'''(\eta )+(1+\lambda )f(\eta )f''(\eta )+2\lambda (1-f'(\eta ))f'(\eta )=0,\quad 0\le \eta <+\infty , \end{aligned}$$

subject to the boundary conditions

2 $$\begin{aligned} f(0)=0,\quad f'(0)=\beta ,\quad f'(+\infty )=1, \end{aligned}$$

where $$\beta \ge 0$$ and $$f'(+\infty ):=\lim \nolimits _{t\rightarrow +\infty }f'(t)$$.

The nonlinear BVP (1–2) with $\beta =0$ is studied (Aly et al. 2003; Nazar et al. 2004) and comes from the study of a plane mixed convection boundary-layer flow near a semi-infinite vertical surface, with a prescribed power law of the distance from the leading edge for the temperature. About BVP (1–2), there have existed some interesting results about the problem above. For example, there admits a unique convex solution (i.e., such that $f''(\eta )>0$) for $\lambda >0$ and $0<\beta <1$ (Brighi and Hoernel 2006); also there admits a unique concave solution (i.e., such that $f''(\eta )<0$) for $\lambda >0$ and $\beta >1$. It is unfortunate that they did not consider the case of $\lambda \le 0$ and few results are available for $\lambda \le 0$.

Current numerical analysis is an important technique for the solution of the Falkner–Skan equation. One key problem for numerical technique is how to deal with the infinite boundary. Early approaches have mainly used shooting or invariant imbedding (Cebeci and Keller 1971; Na 1979). Asaithambi presented an asymptotic condition and truncated the infinite boundary condition by an unknown $\eta _{\infty }$ (Asaithambi 1998, 2004, 2005). Adomian decomposition method was developed to obtain series solutions instead of truncating the infinite boundary (Elgazery 2005; Alizadeh et al. 2009). Yang and Hu (2008) transformed the problem to a singular boundary value problem on finite interval and proposed Galerkin finite element method.

Based on ideas (Yang 2003; Lan and Yang 2008), the purpose of this paper is to transform the problem mentioned above to a singular boundary value problem on a finite interval and develop a finite difference method which is much more effective and simpler than the other existing methods for BVP (1–2), and which requires much less computational effort.

## Transformation formula

Lan and Yang (2008) established the equivalence between the Falkner–Skan equation and a singular integral equation. In this paper, the BVP (1–2) is transformed to a second-order singular boundary value problem, and the solution of BVP (1–2) is characterized by $f''(0)$.

Let $0<\beta <1$ and $f''(\eta )>0 (\eta \ge 0)$, function $t = f'(\eta )$ is strictly increasing in interval $[0,+\infty )$, and its inverse function $\eta =g(t)$ exits and strictly increases in interval $[\beta ,1)$. Then we have $g(\beta )=0, g(1-0)=+\infty$, and

3 $$\begin{aligned} t=f'(g(t)), \quad t\in [\beta ,1). \end{aligned}$$

Differentiating Eq. (3) with respect to *t* yields

4 $$\begin{aligned} w(t):=f''(g(t))=\frac{1}{g'(t)},\quad \beta \le t <1. \end{aligned}$$

Differentiating Eq. (4) with respect to *t* yields

5 $$\begin{aligned} f'''(g(t))=w(t)w'(t),\quad \beta \le t <1. \end{aligned}$$

According to Eq. (3), we obtain $tg'(t)=f'(g(t))g'(t)$, i.e.,

6 $$\begin{aligned} \frac{t}{w(t)}=f'(g(t))g'(t),\quad \beta \le t <1. \end{aligned}$$

Integrating Eq. (6) from $\beta$ to *t* with respect to *s*, we get

7 $$\begin{aligned} \int _{\beta }^{t}\frac{s}{w(s)}ds= \int _{\beta }^{t}f'(g(s))g'(s)ds=f(g(t))-f(g(\beta )). \end{aligned}$$

It follows from $f(g(\beta ))=0$ that

8 $$\begin{aligned} f(g(t))=\int _{\beta }^{t}\frac{s}{w(s)}ds,\quad \beta \le t <1. \end{aligned}$$

Substituting Eqs. (3), (4), (5), and (8) into Eq. (1), we obtain

9 $$\begin{aligned} w(t)w'(t)+(1+\lambda )w(t)\int _{\beta }^{t} \frac{s}{w(s)}ds+2\lambda (1-t)t=0,\quad \beta \le t <1. \end{aligned}$$

Differentiating Eq. (9), we have

10 $$\begin{aligned} w''(t)=\frac{(3\lambda -1)t-2\lambda }{w(t)}+ \frac{2\lambda (1-t)tw'(t)}{w^{2}(t)},\quad \beta \le t <1. \end{aligned}$$

and

11 $$\begin{aligned} w'(\beta )=\frac{-2\lambda (1-\beta )\beta }{w(\beta )}. \end{aligned}$$

On the other hand, according to Eq. (4) and boundary condition $f'(+\infty )=1$, we could obtain boundary condition

12 $$\begin{aligned} w(1)=0. \end{aligned}$$

## Numerical solutions of boundary value problem

Equation (10) can be changed to the following equivalent form

13 $$\begin{aligned} w''(t)w^2(t)+2\lambda {(t-1)tw'(t)}+[(1-3\lambda )t+2\lambda ]w(t)=0,\quad t\in [\beta ,1), \end{aligned}$$

subject to the boundary conditions

14 $$\begin{aligned} w(1)& = {} 0, \end{aligned}$$

15 $$\begin{aligned} w'(\beta )& = {} \frac{\;-2\lambda (1-\beta )\beta \;}{\;w(\beta )\;}. \end{aligned}$$

In this paper, the numerical solution of Eq. (13) with boundary conditions (14, 15) is based on the the finite difference method. The interval $[\beta , 1]$ is divided into *N* subintervals with step size $h=\frac{\;1-\beta \;}{\;N\;}$, and define $t_{j}=\beta +jh$ for $j=0, 1, \ldots , N$. Let $w_j$ denotes the values of $w(t_{j})$ for $j=0, 1, \ldots , N$. Let $t=t_{j}$, the finite difference formulation of Eq. (13) writes as

16 $$\begin{aligned} \frac{\;w_{j+1}-2w_{j}+w_{j-1}\;}{\;h^2\;}w^2_{j} + 2\lambda (t_{j}-1)t_{j}\frac{\;w_{j+1}-w_{j-1}\;}{\;2h\;}+ \big [(1-3\lambda )t_{j}+2\lambda \big ]w_{j}=0, \end{aligned}$$

for $j=1, 2, \ldots , N-1$. The boundary condition (14) corresponds to

17 $$\begin{aligned} w_{N}=0. \end{aligned}$$

And the discretization of boundary condition (15) reads as

18 $$\begin{aligned} \frac{\;w_{1}-w_{0}\;}{\;h\;}w_{0} +2\lambda (1-\beta )\beta =0. \end{aligned}$$

The discretization formulation (16–18) is a nonlinear equation system, so Newton iteration method is recommended to solve approximate solutions. We now proceed to describe the iterative process for the solution of the nonlinear system (16–18). Let ${\mathbf{w}}^{T}=[w_{0}\quad \cdots \quad w_{N}]$, and

19 $$\begin{aligned} {\mathbf{H}}({\mathbf{w}};\lambda )=\left[ \begin{array}{ll} H_{0}({\mathbf{w}};\lambda )\\ \qquad \vdots \\ H_{N-1}({\mathbf{w}};\lambda ) \end{array}\right] , \end{aligned}$$

where

20 $$\begin{aligned} H_{0}({\mathbf{w}};\lambda )=w_{0}w_{1}-w^2_{0}+2h\lambda (1-\beta )\beta , \end{aligned}$$

and

21 $$\begin{aligned} H_{j}({\mathbf{w}};\lambda )=(w_{j+1}-2w_{j}+w_{j-1})w^2_{j} +\lambda {h}(t_{j}-1)t_{j}(w_{j+1}-w_{j-1})+ \big [(1-3\lambda )t_{j}+2\lambda \big ]h^2 w_{j}, \end{aligned}$$

for $$j=1, 2, \ldots , N-1$$.

The solving Eqs. (16–18) is equivalent to solving the system described by

22 $$\begin{aligned} {\mathbf{H}}({\mathbf{w}};\lambda )={\mathbf 0} . \end{aligned}$$

Newton’s iteration method is recommended to solve nonlinear system (22). Given $\lambda$ and initial values $w_{j}^{0}, j=0,1,2,\ldots , N$, the *k*-*th* Newton’s iterates ${\mathbf{w} }^{k}=[w_{0}^k,w_{1}^k, \ldots \quad w_{N}^k]^T,k=1,2,\ldots ,$ can be obtained by solving system (22). Newton’s method for the solution of Eq. (22) proceeds to yield subsequent iterates for **w** as

23 $$\begin{aligned} {\mathbf{w}}^{k+1}={\mathbf{w}}^{k}+\triangle {\mathbf{w}}^{k}, \end{aligned}$$

where $\triangle {\mathbf{w}}^{k}$ satisfies the equation

24 $$\begin{aligned} {\mathbf{J}}_{{\mathbf{H}}}({\mathbf{w}})\triangle {\mathbf{w}}^{k}=-{\mathbf{H}}({\mathbf{w}}^k;\lambda ). \end{aligned}$$

The iterative process described by Eqs. (23, 24) may be repeated in succession until $\Vert \triangle {\mathbf{w}}^{k}\Vert _{\infty }<\varepsilon$ for some prescribed error tolerance $\varepsilon$.

The algorithm is then given as:

Step 1. Input the values $\lambda$, number of subintervals N and stopping condition $\varepsilon$
Step 2. Initialize $\beta$,$k\leftarrow 0$, step size $h \leftarrow \frac{1-\beta }{N}$ and ${\mathbf{w}}_{N}\leftarrow {\mathbf 0}$,
Step 3. Compute ${\mathbf{w}}^{k}, \triangle {\mathbf{w}}^{k}$ by Eqs. (23, 24); $k\leftarrow k+1$
Step 4. Repeat through step 3 until $\Vert \triangle {\mathbf{w}}^{k}\Vert _{\infty }<\varepsilon$ is satisfied.

## Results and discussion

The Falkner–Skan equation has two parameters $\beta$ and $\lambda$, and Aly et al. (2003) obtained some numerical solution for various $\beta$ and $\lambda$. Also, the numerical solutions of the equation have been simulated by using Galerkin finite element methods for various values of $\beta$ and $\lambda$ (Yang and Hu 2008). In order to demonstrate the reliability and efficiency of the proposed theory. The numerical results have been obtained by solving the boundary value problems (13–15) with different parameters $\lambda$ and $\beta$. And comparison of the accuracy for calculation $f''(0)(=w(\beta ))$ is made between our method and Galerkin finite element method proposed in (Yang and Hu 2008), the errors are simulated and shown in Table 1. In numerical simulation, we choose $h=10^{-3}$ and $\varepsilon = 10^{-10}$, respectively. By virtue of equivalent Eqs. (13–15), we can obtained the numerical solution of $f''(0)(= w(\beta ))= 0.4695998$.

**Table 1:** Error results of finite difference method and Galerkin finite element method

$\beta$	$\lambda$					
	$-$0.10	$-$0.15	$-$0.18	$-$0.20	$-$0.25	$-$0.30
0	$2.985\times {10^{-6}}$	$3.433\times {10^{-6}}$	$3.780\times {10^{-6}}$	$4.051\times {10^{-6}}$	$4.892\times {10^{-6}}$	$5.427\times {10^{-6}}$
0.1	$3.010\times {10^{-6}}$	$3.465\times {10^{-6}}$	$3.818\times {10^{-6}}$	$4.096\times {10^{-6}}$	$4.976\times {10^{-6}}$	$5.882\times {10^{-6}}$
0.2	$3.074\times {10^{-6}}$	$3.543\times {10^{-6}}$	$3.910\times {10^{-6}}$	$4.202\times {10^{-6}}$	$5.167\times {10^{-6}}$	$6.962\times {10^{-6}}$
0.3	$3.174\times {10^{-6}}$	$3.664\times {10^{-6}}$	$4.051\times {10^{-6}}$	$4.364\times {10^{-6}}$	$5.452\times {10^{-6}}$	$8.674\times {10^{-5}}$
0.5	$3.516\times {10^{-6}}$	$4.076\times {10^{-6}}$	$4.532\times {10^{-6}}$	$4.913\times {10^{-6}}$	$6.419\times {10^{-6}}$	$1.540\times {10^{-5}}$
0.7	$4.270\times {10^{-6}}$	$4.993\times {10^{-6}}$	$5.609\times {10^{-6}}$	$6.151\times {10^{-6}}$	$8.662\times {10^{-6}}$	$4.706\times {10^{-5}}$
0.9	$7.320\times {10^{-6}}$	$8.807\times {10^{-6}}$	$1.021\times {10^{-5}}$	$1.157\times {10^{-5}}$	$1.993\times {10^{-5}}$	$1.303\times {10^{-4}}$

**Fig. 1:**
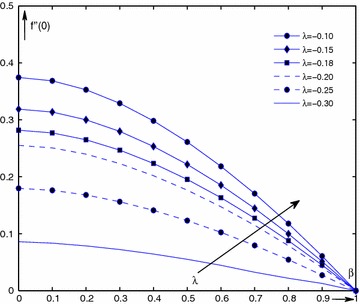
Curve of $f''(0)({=}\,w(\beta ))$ as a function of $\beta$ at interval [0,1] for $\lambda = -0.30, -0.25, -0.20, -0.18, -0.15, -0.10$

It can be seen from Fig. 1, where $f''(0)(=w(\beta ))$ is plotted as a function of $\beta$ in the range of $0\le \beta \le 1$, curves are drawn for value $\lambda = -0.30, -0.25, -0.20, -0.18, -0.15, -0.10$. It is also shown that $f''(0)(=w(\beta ))$ changes smoothly with $\beta$. As $\lambda$ increases, the results also increase in the range of $0\le \beta \le 1$.

**Fig. 2:**
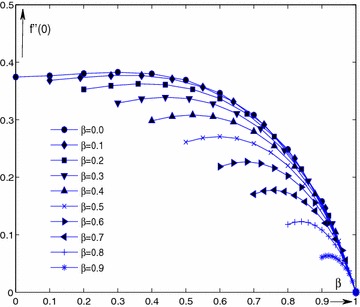
Ten branches of solution $f''(0)({=}\,w(\beta ))$ at interval $[\beta$,1] for $\lambda =-0.1$ and $\beta = 0.0, 0.1, 0.2, 0.3, 0.4, 0.5, 0.6, 0.7, 0.8, 0.9$

Figure 2 shows the characteristics of numerical solutions $f''(0)(=w(\beta ))$ for $\beta =$ 0.0–0.9 by solving the boundary value problems (13–15). The solutions indicate that $f''(0)(=w(\beta ))$ decreases with increasing of the parameter $\beta$, i.e., $f''(0)(=w(\beta ))$ is a decrease function of parameter $\beta$. For each fixed value of $\lambda$, solution of $f''(0)(=w(\beta ))$ decreases with increase of $\beta$ in the range of [0, 1 ], and especially, when $\beta = 0$ and $\lambda = 0$, the classical Balasis solution is obtained (Aly et al. 2003).

Because the Eq. (13) is a second-order boundary value problem, the amount of computational effort used by finite difference method is significantly less than the other numerical methods of the third-order differential equation which essentially solve two or more initial value problems during each iteration (Asaithambi 2004). In general, the numerical simulation shows that the initial guess for ${\mathbf{w}}^{0}$ could be far away from the exact value. For each fixed value of ${\mathbf{w}}^{0}$, the method in this paper required 2–6 iterations in order to solve system (22) to the desired accuracy.

## Conclusions

In this work, we have demonstrated the effectiveness of the finite difference method to Falkner–Skan equation. Applying equivalent transformation to Falkner–Skan equation, a third-order boundary value problem in infinite interval is transformed into a second-order boundary value problem in finite interval. By using finite difference method and Newton’s iteration approximation, the numerical solution have been calculated.

The results of comparison studied in this paper indicate that, the values of the Newton’s iteration for $f''(0)(=w(\beta ))$ are in excellent agreement with those results obtained by previous authors. Therefore, the method presented in this work shows its validity and great potential for the solution of Falkner–Skan equations arising in science and engineering .
